# Environmental Factors Influencing Phyllosphere Bacterial Communities in Giant Pandas’ Staple Food Bamboos

**DOI:** 10.3389/fmicb.2021.748141

**Published:** 2021-11-03

**Authors:** Juejie Long, Wei Luo, Jianmei Xie, Yuan Yuan, Jia Wang, Liwen Kang, Yi Li, Zejun Zhang, Mingsheng Hong

**Affiliations:** ^1^Liziping Giant Panda’s Ecology and Conservation Observation and Research Station of Sichuan Province (Science and Technology Department of Sichuan Province), China West Normal University, Nanchong, China; ^2^Key Laboratory of Southwest China Wildlife Resources Conservation (Ministry of Education), China West Normal University, Nanchong, China; ^3^Liziping National Nature Reserve Administration, Ya’an, China

**Keywords:** giant panda, phyllosphere bacteria, influencing factors, microbial diversity, metagenome, bamboo

## Abstract

The giant panda has developed a series of evolutionary strategies to adapt to a bamboo diet. The abundance and diversity of the phyllosphere microbiome change dramatically depending on the season, host species, location, etc., which may, in turn, affect the growth and health of host plants. However, few studies have investigated the factors that influence phyllosphere bacteria in bamboo, a staple food source of the giant panda. Amplicon sequencing of the 16S rRNA gene was used to explore the abundance and diversity of phyllosphere bacteria in three bamboo species (*Arundinaria spanostachya*, *Yushania lineolate*, and *Fargesia ferax*) over different seasons (spring vs. autumn), elevation, distance from water, etc., in Liziping National Nature Reserve (Liziping NR), China. And whole-genome shotgun sequencing uncovered the differences in biological functions (KEGG and Carbohydrate-Active enzymes functions) of *A. spanostachya* phyllosphere bacteria between spring and autumn. The results showed that the abundance and diversity of *F. ferax* phyllosphere bacteria were greater than that of the other two bamboo species in both seasons. And three kinds of bamboo phyllosphere bacteria in autumn were significantly higher than in spring. The season was a more important factor than host bamboo species in determining the community structure of phyllosphere bacteria based on the (un)weighted UniFrac distance matrix. The composition, diversity, and community structure of phyllosphere bacteria in bamboo were primarily affected by the season, species, altitude, tree layer, and shrub layer. Different bacterial communities perform different functions in different bamboo species, and long-term low temperatures may shape more varied and complex KEGG and Carbohydrate-Active enzymes functions in spring. Our study presented a deeper understanding of factors influencing the bacterial community in the bamboo phyllosphere. These integrated results offer an original insight into bamboo, which can provide a reference for the restoration and management of giant panda bamboo food resources in the Xiaoxiangling mountains.

## Introduction

Large numbers of different kinds of microorganisms within the phyllosphere constitute a stable and complex micro-ecosystem (Mei, 1991; Li, 1998; Shi and Zhang, 2007; Vorholt, 2012). Different hosts have different microbial communities (Yadav et al., 2008; Leveau, 2015), including bacteria, archaea, fungi, and protists (Newton et al., 2010). Bacteria are the most abundant microorganism in the phyllosphere (Lindow and Brandl, 2003) and Proteobacteria are the dominant phylum (Delmotte et al., 2009).

Numerous environmental factors determine phyllosphere community composition, such as plant species, temperature, humidity, nutrient availability (on the plant surface), sun/UV exposure levels, and even the underlying soil geochemistry (Lindow and Brandl, 2003; Leveau, 2015). Ecological factors and plant attributes, e.g., wood density, leaf mass per unit area, and leaf nitrogen and phosphorus concentration, may also affect the microbial community structure in the phyllosphere (Steven et al., 2014).

The phyllosphere microbial community represents a wide range of primitive symbiotic relationships (Fedorov et al., 2011; Partida and Martin, 2011). Phyllosphere microbiota can affect plant yield (e.g., via nutrients or hormones) (Gourion et al., 2006; Reed et al., 2010) and plant health in a variety of ways (Balint-Kurti et al., 2010; Gerd et al., 2011). For example, *Beauveria* fungus on rice leaf can protect rice enzyme activity and is an environmentally friendly microbe (Du et al., 2014). Some phyllosphere microbes are beneficial to plants via nitrogen fixation (Leveau, 2015), growth promotion (Bringel and Couée, 2015), acting as barriers against pathogen invasion, and decomposition of residual pesticides (Venkatachalam et al., 2016).

Bamboo is a perennial evergreen plant belonging to the Gramineae family and Bambusoideae subfamily and is an important forest resource all over the world. It has the characteristics of being widely distributed, fast-growing and high-yield, and strong regeneration ability. Therefore, it has considerable economic, ecological, and social benefits (Shanmughavel and Francis, 1997; Gupta and Kumar, 2008; Chang and Chiu, 2015; Zhang and Xue, 2018). Bamboo leaves are an important habitat for many microorganisms, with microbial abundance and diversity acting as an indicator of forest health. However, few studies have investigated the environmental factors that influence phyllosphere bacterial composition in bamboo. Using the traditional cultivation method, Zhang et al. (2014) found that the composition of the phyllosphere microbial community differed between bamboo species and seasons. The species composition and frequency of leaf endophytic bacteria and fungi have also been found to differ between bamboo species (Helander et al., 2013), while endophytic fungi from fish-scale bamboo (*Phyllostachys heteroclada*) differs between the branch and leaf tissue (Zhou et al., 2017). Significant differences in bacterial richness and diversity were also observed between different bamboo species using high throughput amplicon sequencing (Jin et al., 2020). However, there is a lack of research on what factors influence the composition and diversity in the bamboo phyllosphere microbiome.

The giant panda (*Ailuropoda melanoleuca*) belongs to a carnivorous clade (Wei et al., 1999), and yet has an exclusively herbivorous diet and specializes in the consumption of bamboo leaves throughout the year (Hu et al., 1985; Zhao et al., 2013). In the long evolutionary process, the giant panda developed a series of foraging strategies to adapt to a bamboo diet, such as seasonal vertical migration, selection of habitat, and feeding point (Hong et al., 2015, 2016). However, in the different seasons of different mountain systems, the diet of giant pandas does vary. For example, in the Qinling mountains, giant pandas mainly feed on bamboo leaves during the non-bamboo shoot seasons (Pan, 2001; Wu et al., 2017), while bamboo leaves of the Xiaoxiangling mountains account for more than half of the panda feces in summer and autumn (Wei et al., 1999). However, leaves usually contribute to intestinal diseases of giant pandas in captivity, and bacterial infections are an important cause of intestinal diseases. Previous research has shown that *Escherichia coli* and *Klebsiella pneumoniae* could cause giant pandas’ diarrhea and septicemia (Zhang and Chen, 1997; Xiong et al., 1999). However, there are few studies concentrating on the phyllosphere microbiome in the bamboo species foraged by wild giant pandas.

In this study, we investigate the phyllosphere bacterial community of bamboo species frequently used as a food source by giant pandas, using Next Generation Sequencing Technology (NGS) in Liziping National Nature Reserve (Liziping NR), Sichuan, China. The specific goals include (i) comparing interspecies differences and seasonal diversity of phyllosphere bacterial communities in bamboo; (ii) exploring the ecological factors that influence the phyllosphere bacteria community changes; and (iii) revealing the functional differences of phyllosphere bacterial communities among different bamboo species/different seasons. Our integrated results can provide a reference for the restoration and management of giant panda bamboo food resources in the Xiaoxiangling mountains.

## Materials and Methods

### Study Area

Liziping NR is located in Shimian County, Sichuan Province, China, and is in the middle and upper reaches of the Dadu River, on the southwestern edge of the Sichuan Basin and southeast of Gongga Mountain (E102°10′33′′-E102°29′07′′, N28°51′02′′ -N29°08′42′′) (Hong et al., 2015). The reserve covers an area of 47,940 km^2^ and ranges from 1,330 to 4,550 m above sea level, with uneven ridges and narrow valleys. The annual average temperature and rainfall are 11.7–14.4°C and 800–1250 mm, respectively (Xei and Hong, 2020). As the altitude increases, the vegetation in the reserve transitions from evergreen broad-leaved forest to deciduous broad-leaved forest, then coniferous and broad-leaved mixed forest, coniferous forest to alpine thickets, and alpine rocky beech. According to the Fourth National Survey of giant pandas, there were 22 giant pandas in the reserve in 2015 (Sichuan Forestry Bureau, 2015). To strengthen the small isolated populations of giant pandas, the Chinese government has initiated the Captive Giant Panda Release Program (CGPRP). To date, one giant panda rescued from the wild and nine giant pandas from captivity have been released into this nature reserve (Hong et al., 2012). There are seven bamboo species (three genera) consumed by the giant panda within the Xiaoxiangling Mountains. The species with the largest distribution is *Arundinaria spanostachya*, which accounts for 38.08% of the total area of giant panda feeding bamboos in these mountains. Next is *Yushania lineolate* and *Fargesia ferax*, which account for 28.02 and 12.48% of the giant panda’s feeding bamboo, respectively (Sichuan Forestry Bureau. 2015). *A. spanostachya* mainly grows above 2,500 m a.s.l, while *Y. lineolate* and *F. ferax* occur below 2,800 m a.s.l. Giant pandas in this reserve prefer to eat *A. spanostachya* throughout the whole year, some *Y. lineolate* in winter, and occasionally *F. ferax* (Hong et al., 2015, 2016; Xei and Hong, 2020).

### Experimental Design

We conducted wild surveys and sampling during May (spring) and October (autumn) in 2020. First, we set up four transects in *A. spanostachya*, *Y. lineolate*, and *F. ferax* bamboo forests, respectively. The transects were set from low altitude to high altitude, and the distance between them was no less than 200 m. Secondly, we set up three to five survey plots (20 m × 20 m) in each transect, with the altitude distance of adjacent survey plots on the same transect no less than 50 m. We then recorded and measured bamboo species, latitude and longitude, altitude, and other related variables in the tree and shrub layer. Moreover, one bamboo plot (1 m × 1 m) was set up in the center of each survey plot, and another two bamboo plots (1 m × 1 m) were set up east and south, 5 m from the center point of each survey plot. Finally, the related variables within the bamboo layer were also measured and recorded (Supplementary Table 1).

### Sample Collection and DNA Extraction

In each survey plot, one mixed bamboo leaf sample (not less than 200 g) was collected with sterile gloves, immediately transported to the laboratory (less than 2 h), and stored at −20°C for further processing within 48 h. Each 200 g sample was aseptically transferred into a Ziplock bag (24 cm × 35 cm) containing 200 ml sterile precooled TE-buffer (10 mM Tris, 1 mM EDTA, and pH 7.5) supplemented with 0.05% Tween-80 (Hong et al., 2017). Leaf surfaces were washed to collect the microbial population by 5 min of shaking, vortexing, and sonication of each sample in the TE-buffer, with the Ziplock bag kept in ice water (∼4°C) for each processing step (Hong et al., 2017). The cell suspension was separated from the leaf material by filtration through three-layer sterile nylon mesh. Sonication was performed at a frequency of 40 kHz in an ultrasonic cleaning bath (Shanghai Kudos Instrument Co., Shanghai, China) to dislodge the microbes from the leaf surface. Following filtration, cell suspensions were placed in four 50 ml tubes/sample, and cells were pelleted using centrifugation at 2,000 × *g* for 15 min at 4°C. Cell pellets from multiple tubes were pooled into 2.0-ml reaction tubes and washed twice with TE-buffer with Tween-80. Cell pellets were immediately frozen at −80°C until DNA extraction.

The DNA extraction was performed using the E.Z.N.A.TM Soil DNA Kit (Omega, Norcross, GA, United States) as described with slight modifications. Frozen cell pellets were resuspended in 1 ml of kit-supplied SLX Mlus buffer with 500 mg of glass beads, and cell lysis was performed at 65 Hz for 90 s. The cell debris suspension was immediately processed following the instructions in the kit manual. Finally, the total DNA was obtained from the column by two sequential elutions with 50 μl elution buffer.

### 16S rRNA Gene V3–V4 Amplification, Quantification, and Sequencing

The sequencing of the V3–V4 hypervariable region of the 16S rRNA gene was performed for bacterial identification (Caporaso et al., 2011; Lundberg et al., 2013). Thirty-five cycles of polymerase chain reaction (PCR) amplification of the target marker genes were performed. Error-correcting 12-bp barcoded primers specific to each sample were used to permit multiplexing of samples. Polymerase chain reaction products from all samples were quantified using the PicoGreen dsDNA assay and pooled together in equimolar concentrations. Each library was submitted to Mega Biotech on the Illumina MiSeq PE300 platform.

### Sequence Data Analysis

The raw 16S rRNA gene sequencing reads were demultiplexed, quality-filtered by fastp version 0.20.0 (Chen et al., 2018), and merged by FLASH version 1.2.7 (Magoč and Salzberg, 2011) with the following criteria: (i) the 300 bp reads were truncated at any site receiving an average quality score of <20 over a 50 bp sliding window, and the truncated reads shorter than 50 bp were discarded. Reads containing ambiguous characters were also discarded; (ii) only overlapping sequences longer than 10 bp were assembled according to their overlapped sequence. The maximum mismatch ratio of the overlap region was 0.2. Reads that could not be assembled were discarded; (iii) samples were distinguished according to the barcode and primers. The sequence direction was adjusted using exact barcode matching with two nucleotide mismatches in primer matching.

Operational taxonomic units (OTUs) with 97% similarity cut-off (Stackebrandt and Goebel, 1994; Edgar, 2013) were clustered using UPARSE version 7.1, and chimeric sequences were identified and removed. The taxonomy of each OTU representative sequence was analyzed by RDP Classifier version 2.2 (Wang et al., 2007) against the 16S rRNA database (e.g., Silva v138) using a confidence threshold of 0.7. Non-target sequences, including mitochondrial and chloroplast sequences, were also removed by QIIME from the final OTU data set. To better convey the biological information in these samples, the average relative abundance of the bacterial community was visualized by bar chart at the level of phylum and genus.

### Statistical Analysis

First, Two-way ANOVA (species × season) were used to exam the differences in bacterial abundance between dominant bacteria abundance at both phylum and genus levels. We used mothur software (version v.1.30.1) to calculate the Sobs and Shannon indices for each sample, to estimate the species abundance and diversity of phyllosphere bacteria between the different bamboo species and seasons, and used one-way ANOVA to identify any significant differences. We then used PCoA based on the weighted UniFrac and unweighted UniFrac method distance matrix to evaluate the differences in the microbial community structure between the bamboo species and seasons. We also used the permutational MANOVA (PERMANOVA) to analyze the effect of different bamboo species and seasons on the phyllosphere bacterial community, based on Bray–Curtis distance matrices with a substitution test to analyze the statistical significance of the division.

Second, Spearman’s correlation heatmap analysis was performed to examine the relationship between the relative abundance of bacterial taxa and the environmental factors described in Supplementary Table 1. To explore the spatial association between each environmental factor and phyllosphere bacterial community, Mantel tests were performed to examine the spatial correlation between each environmental factor distance matrix and phyllosphere bacterial UniFrac distance matrix based on OUT level. Redundancy analysis (RDA) was used to detect the relationship between environmental factors, samples and microflora, or directly detect the relationship between two pairs. Linear regression was used to evaluate the relationship between environmental factors and the results of either Alpha (Sobs and Shannon indices) or Beta diversity analysis (Bray–Curtis distance).

Finally, the PICRUSt prediction was used to predict the functional composition of all phyllosphere bacteria in the different bamboo species. The greengene id corresponding to each OTU, the COG, and KEGG functions of the OTU were annotated to obtain the function level of COG and KEGG, and the abundance information for each function in different samples.

### Metagenome Sequencing and Analysis

The raw reads from metagenome sequencing were used to generate clean reads by removing adaptor sequences, trimming and removing low-quality reads (reads with N bases, a minimum length threshold of 50 bp, and a minimum quality threshold of 20) using the fastp^1^ (Chen et al., 2018) (version 0.20.0) on the free online platform of Majorbio Cloud Platform (cloud.majorbio.com). The clean reads were mapped to the host reference genome using BWA^2^ (Li and Durbin, 2009) (version 0.7.9a) to identify and remove the host-originated reads. These high-quality reads were then assembled to contigs using MEGAHIT (Li et al., 2015) (parameters: kmer_min = 47, kmer_max = 97, step = 10)^3^ (version 1.1.2), which makes use of succinct de Bruijn graphs. Contigs with the length being or over 300 bp were selected as the final assembling result. Open reading frames (ORFs) in contigs were identified using MetaGene (Noguchi et al., 2006). The predicted ORFs with a length equal to or greater than 100 bp were retrieved and translated into amino acid sequences using the NCBI translation table^4^.

A non-redundant gene catalog was constructed using CD-HIT^5^ (Fu et al., 2012) (version 4.6.1) with 90% sequence identity and 90% coverage. Reads after quality control were mapped to the non-redundant gene catalog with 95% identity using SOAPaligner (Li et al., 2008) (version 2.21), and gene abundance in each sample was evaluated. Representative sequences of non-redundant gene catalog were annotated based on the NCBI NR database using blastp as implemented in DIAMOND v0.9.19 with an e-value cutoff of 1e-5 using Diamond^6^ (Buchfink et al., 2015) (version 0.8.35) for taxonomic annotations. For functional analyses, KEGG annotation was conducted using Diamond (Buchfink et al., 2015) (see Text Footnote 8, version 0.8.35) against the Kyoto Encyclopedia of Genes and Genomes database^7^ (version 94.2) with an *e*-value cutoff of 1e-5. Carbohydrate-active enzymes annotation was conducted using hmmscan against CAZy database^8^ with an e-value cutoff of 1e-5. Abundant different features of KEGG Level 2 and CAZyme were determined using linear discriminant analysis (LDA) effect size (LEfSe) and setting 2 as the threshold on the logarithmic LDA score (Segata et al., 2011).

## Results

### Community Composition Among Different Bamboo Species in Spring and Autumn

**FIGURE 1:**
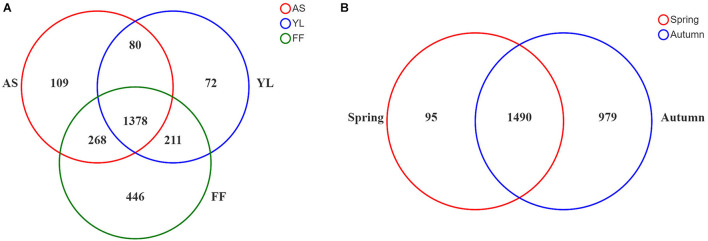
Venn diagram showing overlap/non-overlap of phyllosphere bacteria OTUs among three bamboo species **(A)** and two seasons **(B)**. AS, *A. spanostachya*; YL, *Y. lineolate*; FF, *F. ferax*.

Up to 19 samples of each bamboo species were collected in each study season (Table 1). After removing the mitochondrial and chloroplast sequences, a total of 1,233,917 effective target 16S rRNA reads were obtained. The average sequence of each sample was 12,217 ± 3,037. Clustered by 97% similarity, all samples had a total of 2,564 OTUs. A total of 1,378 OTUs were shared between the three bamboo species, and the bacterial OTUs of each in autumn were significantly higher than in spring (Figure 1 and Supplementary Figure 1). *Fargesia ferax* had a higher number of OTUs than the other two species in both seasons (Figure 1A).

**TABLE 1:** Statistics table of each sample and sequence (AS, *A. spanostachya*; YL, *Y. lineolate*; and FF, *F. ferax*) (Mean ± *SD*).

Season	Spring			Autumn		
Bamboo species	AS	YL	FF	AS	YL	FF
Samples	17	17	16	19	16	16
Total number of sequences	41,426 ± 5,012	45,313 ± 5,317	43,209 ± 4,450	53,715 ± 4,620	51,920 ± 2,923	52,921 ± 6,892
Total number of effective sequences	23,149 ± 2,291	24,619 ± 3,70125	20,013 ± 3,572	24,799 ± 1,453	25,286 ± 797	24,098 ± 2,170

**FIGURE 2:**
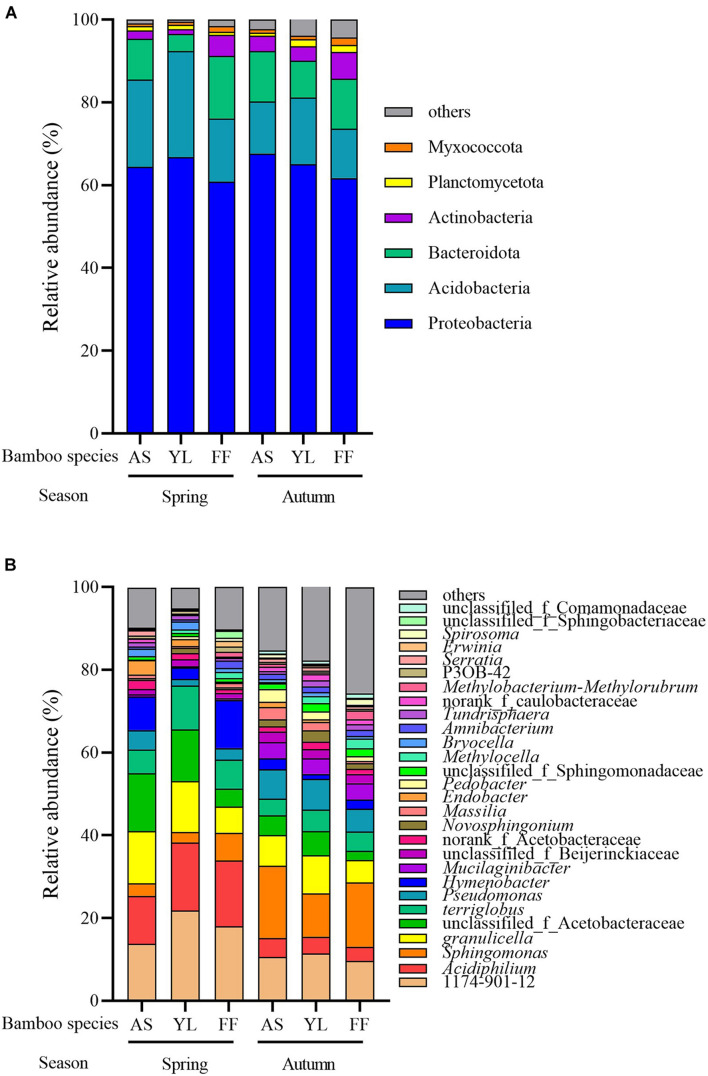
Relative abundance of different phyllosphere bacterial phyla **(A)** and genus **(B)** among *A. spanostachya* (AS), *Y. lineolate* (YL), and *F. ferax* (FF) in spring and autumn.

After annotating the representative sequence of OTU with species clustering, all bacterial OTUs were sorted into 24 phyla and 614 genera. The most dominant phylum abundance was Proteobacteria, accounting for over 60% of the bacterial relative abundance observed across all samples (Figure 2A). The remaining bacteria were distributed among the phyla Acidobacteria, Bacteroidota, Actinobacteria, Planctomycetota, Myxococcota, and others. In spring, the relative abundance of phyla Bacteroidota, Actinobacteriota, and Myxococcota in the *F. ferax* phyllosphere were significantly higher than that of *A. spanostachya* and *Y. lineolate*. However, the relative abundance of phylum Acidobacteriota in the *F. ferax* phyllosphere was significantly lower than that of *A. spanostachya* and *Y. lineolate*. In autumn, the relative abundance of phyla Actinobacteriota and Myxococcota in the *F. ferax* phyllosphere were significantly higher than that of *A. spanostachya* and *Y. lineolate*. The relative abundance of phyla Bacteroidota in *Y. lineolate* and Planctomycetota in the *A. spanostachya* phyllosphere was significantly lower than that of the other two bamboo species. No difference was found in the relative abundance of dominant phyla in the *F. ferax* phyllosphere between seasons, except Planctomycetota. The relative abundance of phylum Acidobacteriota in the *A. spanostachya* and *Y. lineolate* phyllospheres were lower in autumn than spring, and the relative abundance of the phyla Bacteroidota, Actinobacteriota, and Myxococcota all increased from spring to autumn (Table 2).

**TABLE 2:** Two-way ANOVA (species × season) examining the differences in bacterial abundance between dominant bacteria abundance at both phylum and genus levels.

Phylum	Control variables	*F*	*p*
Proteobacteria	Season	0.078	0.78
	Species	4.784	0.01
	Season * species	0.557	0.575
Acidobacteriota	Season	19.945	<0.001
	Species	7.807	0.001
	Season * species	1.314	0.274
Bacteroidota	Season	1.403	0.239
	Species	14.133	<0.001
	Season * species	4.324	0.016
Actinobacteriota	Season	8.766	0.004
	Species	12.098	<0.001
	Season * species	0.198	0.821
Planctomycetota	Season	8.632	0.004
	Species	2.416	0.095
	Season * species	5.785	0.004
Myxococcota	Season	3.789	0.055
	Species	14.28	<0.001
	Season * species	0.184	0.832

**Genu**	**Control variable**	***F***	***p***

1174-901-12	Season	50.999	<0.001
	Species	7.142	0.001
	Season * species	4.495	0.014
*Sphingomonas*	Season	260.655	<0.001
	Species	18.622	<0.001
	Season * species	10.184	<0.001
*Acidiphilium*	Season	185.807	<0.001
	Species	3.022	0.053
	Season * species	5.631	0.005
*Granulicella*	Season	14.614	<0.001
	Species	13.925	<0.001
	Season * species	1.952	0.148
Unclassified_f__Acetobacteraceae	Season	98.923	<0.001
	Species	40.713	<0.001
	Season * species	11.878	<0.001
*Terriglobus*	Season	22.237	<0.001
	Species	7.184	0.001
	Season * species	3.103	0.05
*Pseudomonas*	Season	25.055	<0.001
	Species	2.141	0.123
	Season * species	2.384	0.098
*Hymenobacter*	Season	43.704	<0.001
	Species	12.267	<0.001
	Season * species	7.552	0.001

The relative abundances of genera 1174-901-12, *Acidiphilium*, *Sphingomonas*, unclassified_f__Acetobacteraceae, *Terriglobus*, *Granulicella*, *Pseudomonas*, and *Hymenobacter* revealed them to be the dominant bacteria in the phyllosphere of all three bamboo species, and the differences among the top eight total abundances of genera were calculated. In spring, the relative abundance of 1174-901-12, *Acidiphilium*, and *Terriglobus* were highest in the *Y. lineolate* phyllosphere. The relative abundance of *Hymenobacter* and *Sphingomonas* was highest in the *F. ferax* phyllosphere, but the opposite pattern was observed for *Granulicella*. In autumn, the lowest relative abundance of *Acidiphilium* and *Granulicella* existed in the *F. ferax* phyllosphere, and that of *Hymenobacter* and *Sphingomonas* existed in the *Y. lineolate* phyllosphere. For all three bamboo species, similar relative abundance patterns were found for the seven most dominant bacteria genus in each phyllosphere. The relative abundance of 1174-901-12, *Acidiphilium*, *Hymenobacter*, *Granulicella*, and *Terriglobus* decreased from spring to autumn but increased for *Sphingomonas* and *Pseudomonas* (Figure 2B and Table 2).

### Community Diversity Among Different Bamboo Species in Spring and Autumn

**FIGURE 3:**
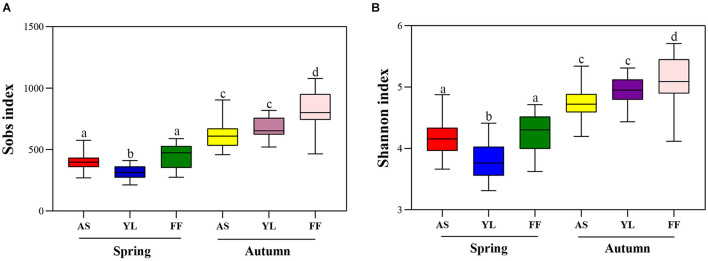
The observed species index (Sobs index) **(A)** and Shannon index **(B)** of phyllosphere bacterial community among *A. spanostachya* (AS), *Y. lineolate* (YL), and *F. ferax* (FF) in spring and autumn. One-way ANOVA was used to compare the significance. Different letters indicate significant differences.

The Sobs and Shannon indices for the bacterial community in the *F. ferax* phyllosphere were higher than that of *A. spanostachya* and *Y. lineolate* in both seasons. However, in spring the Sobs and Shannon indices in *Y. lineolate* were lower than that of *F. ferax* and *A. spanostachya*. The phyllosphere bacterial community of *A. spanostachya* showed the lowest Sobs and Shannon indices in autumn (Figure 3 and Supplementary Table 2). These indices were also found to increase significantly from spring to autumn in all bamboo species.

**FIGURE 4:**
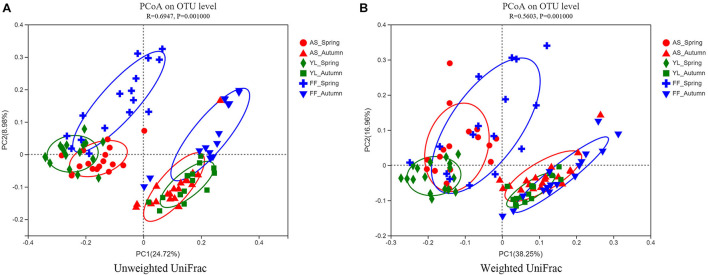
Principal coordinate analysis (PCoA) plots of phyllosphere bacterial communities using the unweighted UniFrac method **(A)** and weighted UniFrac method **(B)** at OTU level among *A. spanostachya* (AS), *Y. lineolate* (YL), and *F. ferax* (FF) in spring and autumn.

The results of the PCoA revealed that samples were clustered by species and season. Samples of *A. spanostachya* and *Y. lineolate* were also clustered together away from that of *F. ferax* in the same season. The PCoA based on unweighted UniFrac distinguished samples better than that based on weighted UniFrac (Figure 4). The PERMANOVA revealed significant differences in phyllosphere bacterial community based on Bray–Curtis distance matrixes between the three bamboo species in spring and autumn (Table 3).

**TABLE 3:** PERMANOVAs exploring the effects of different bamboo species (AS, *A. spanostachya*; YL, *Y. lineolate*; and FF, *F. ferax*) and seasons (Spring and Autumn) on the phyllosphere bacteria community based on Bray–Curtis distance matrixes.

Grouping factors	SumsOfSqs	MeanSqs	*F*.Model	*R* ^2^	*P*-value
AS (Spring:Autumn)	1.627	1.627	20.805	0.380	0.001
YL (Spring:Autumn)	1.552	1.552	24.229	0.439	0.001
FF (Spring:Autumn)	1.365	1.365	13.486	0.310	0.001
Autumn (AS:YL:FF)	1.089	0.545	6.912	0.227	0.001
Spring (AS:YL:FF)	1.842	0.921	11.025	0.319	0.001

### Ecological Factors Influencing Bamboo Phyllosphere Bacterial Community

**FIGURE 5:**
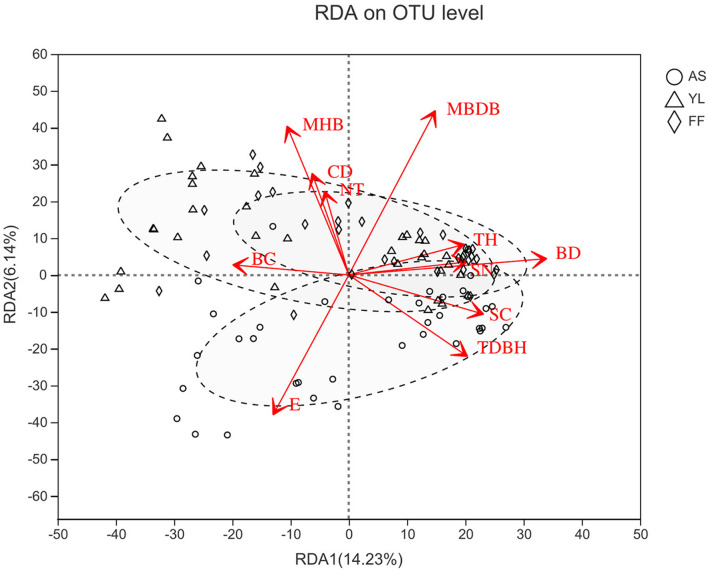
Redundancy analysis (RDA) between environmental factors and phyllosphere bacterial communities at OTU level among *A. spanostachya* (AS), *Y. lineolate* (YL), and *F. ferax* (FF). E, elevation; TH, trees height; TDBH, trees diameter at breast height; SN, shrubs numbers; MHB, mean height of bamboo; MBDB, mean base diameter of bamboo. CD, canopy density; BC, bamboo coverage; BD, bamboo deaths; NT, number of trees.

The results of Mantel analysis revealed that elevation had the greatest impact on the phyllosphere bacterial community (*R* = 0.313, *P* = 0.001). And elevation, distance from water, trees height, trees diameter at breast height, shrubs coverage, shrubs numbers, mean height of bamboo, and mean base diameter of bamboo also had significant impacts on the phyllosphere bacterial community (Supplementary Table 3). Furthermore, RDA analysis showed that the number of trees, canopy density, bamboo coverage, and bamboo deaths also influenced the bacterial community in samples (Figure 5 and Supplementary Table 4). However, distance from water and shrubs coverage had no significant impact on bacterial community in samples (Figure 5 and Supplementary Table 4).

**FIGURE 6:**
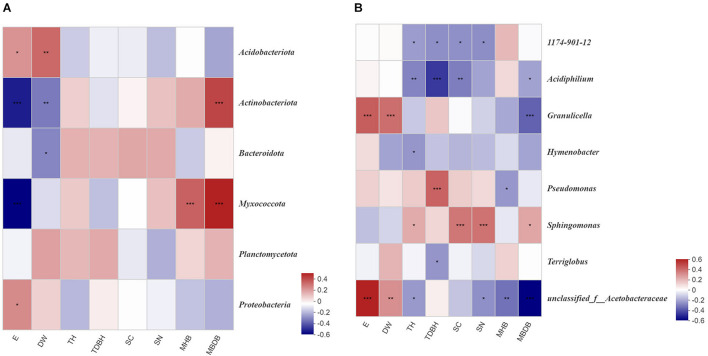
Correlation heatmap of bamboo environmental factors and read numbers at the phylum **(A)** and genus **(B)** level for bacteria. E, elevation; DW, distance from water; TH, trees height; TDBH, trees diameter at breast height; SC, shrubs coverage; SN, shrubs numbers; MHB, mean height of bamboo; MBDB, mean base diameter of bamboo. The color intensity in each panel indicates the relative correlation between bamboo environmental factors and read numbers of each group. *0.01 < *P* < 0.05, **0.001 < *P* < 0.01, ***P < 0.001.

Spearman’s correlation heatmap analysis revealed that Proteobacteria (*P* < 0.05) and Acidobacteria (*P* < 0.05) abundance were positively correlated with elevation (E), while Actinobacteria (*P* < 0.001) and Myxococcota (*P* < 0.001) abundance were negatively correlated with elevation (E). Actinobacteria (*P* < 0.001) and Myxococcota (*P* < 0.001) abundance were positively correlated with the mean base diameter of bamboo (MBDB). Acidobacteria (*P* < 0.01) abundance was positively correlated with distance from water (DW), but Actinobacteria (*P* < 0.01) and Bacteroidota (*P* < 0.05) were negatively correlated with distance from water (DW). Myxococcota (*P* < 0.001) abundance was positively correlated with the mean height of bamboo (MHB) (Figure 6A). At the genus level, 1174-901-12 and *Acidiphilium* were both negatively correlated with tree height (TH), trees diameter at breast height (TDBH), shrub coverage (SC), and shrubs number (SN). Positive correlations were observed between *Sphingomonas* and shrubs number (SN), tree height (TH), and mean base diameter of bamboo (MBDB). *Granulicella* showed significant correlations with elevation (E) and distance from water (DW), but negative correlations with the mean base diameter of bamboo (MBDB) (Figure 6B).

Further linear regression found that elevation (E), bamboo deaths (BD), mean base diameter of bamboo (MBDB), bamboo coverage (BC), and tree height (TH) had a significant impact on the Sobs and Shannon indices (Supplementary Table 5). Elevation (E) had the highest influence on the Sobs index, followed by bamboo deaths (BD), mean base diameter of bamboo (MBDB), bamboo coverage (BC), and tree height (TH). The highest ecological influence factor for the Shannon index and Bray–Curtis distance was bamboo deaths (BD). Furthermore, shrub coverage (SC) and shrubs number (SN) all had a significant impact on bacterial community structure in the bamboo phyllosphere based on Bray–Curtis distance matrixes (Supplementary Table 5).

### Function Differences Among Different Bamboo Species/Different Seasons

**FIGURE 7:**
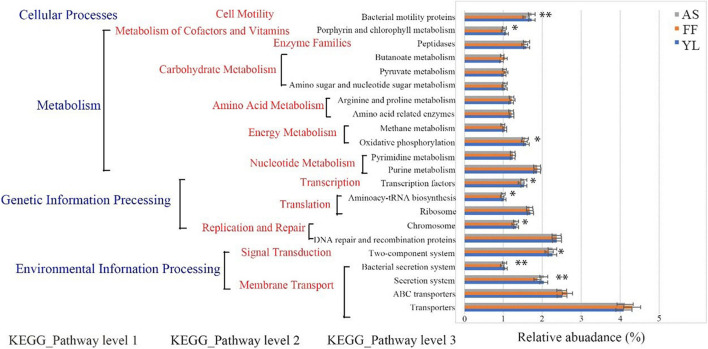
Functional predictions for phyllosphere bacterial communities with significantly different KEGG pathways (*P* < 0.05) for the three bamboo species (AS, FF, and YL). KEGG pathways at Level 1, Level 2, and Level 3 are represented. *0.01 < *P* < 0.05, **0.001 < *P* < 0.01.

All three bamboo phyllosphere bacterial communities had similar COG function classification patterns as generated by PICRUSt in both spring and autumn (Supplementary Figure 2). There were higher relative abundance sequences related to cell wall/membrane/envelope biogenesis, amino acid transport, and metabolism. The relative abundance of transporter function (4.17%) was the highest in all three bamboo species, followed by ABC transporters (2.55%), DNA repair and recombination proteins (2.37%), and two-component systems (2.23%) (Supplementary Figure 2). Prediction software PICRUSt enriched 22 categorizable dominant pathways (relative abundance > 1%) in the level 3 KEGG pathway. Among them, nine pathways had significant differences between bamboo species (*P* < 0.05) (Figure 7 and Supplementary Table 6). It is worth noting that there were significant differences in the relative abundance of the bacterial secretion system, the secretion system, and the two-component system. Oxidative phosphorylation in energy metabolism, porphyrin, and chlorophyll metabolism were also different, and the gene relative abundance for these functions for *F. ferax* was significantly lower than that of the other two species (Figure 7 and Supplementary Table 6).

**FIGURE 8:**
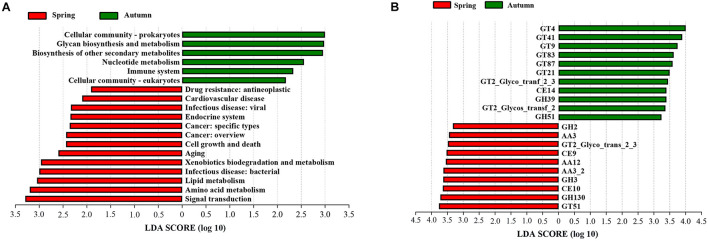
Linear discriminant analysis (LDA) effect size (LEfSe) analysis of KEGG level 2 **(A)** and CAZy **(B)** between *A. spanostachya* in spring and autumn. GT, glycosyl transferases; CE, carbohydrate esterases; GH, glycoside hydrolases; AA, auxiliary activities. LDA > 2.

To explore the functional potential of phyllosphere bacteria in bamboo between different seasons, a total of eight bamboo samples from *A. spanostachya* (spring, four samples; autumn, four samples) were used to perform metagenomic sequencing. After filtering out low-quality and host genomic reads, 129.50 Gb of high-quality reads were obtained, with an average of 16.18 Gb for each sample. In total, 3.91 million contigs and 2,473 million bp of assembly sequence were obtained with an average contig N50 of 5,238 bp (Supplementary Table 7). A non-redundant gene catalog containing 2,206,289 entries was constructed, with an average of 275,786 entries per sample (Supplementary Table 7). The representative sequences of the non-redundant gene catalog were annotated to obtain five domains, and the bacterial domain was further studied in this paper. The metagenomic analysis confirmed 456 KOs, including 44 KEGG Level 2 categories in bacteria. *A. spanostachya* in spring displayed high abundances in KEGG Level 2 categories of signal transduction, amino acid metabolism, lipid metabolism, infectious disease: bacterial, xenobiotics biodegradation and metabolism, aging, cell growth and death, cancer: overview, cancer: specific types, endocrine system, infectious disease: viral, cardiovascular disease, and drug resistance: antineoplastic (Figure 8A), whereas cellular community-eukaryotes, immune system, nucleotide metabolism, biosynthesis of other secondary metabolites, glycan biosynthesis and metabolism, and cellular community-prokaryotes exhibited higher abundance in autumn (Figure 8A). According to the LEfse results of the CAZyme, four families had significantly higher relative abundance in spring, including glycoside hydrolases (GH2, GH3, and GH130), carbohydrate esterases (CE9 and CE10), and glycosyl transferases (GT51 and GT2_Glyco_trans_2_3) and auxiliary activities (AA3, AA12, and AA3_2); interestingly, in autumn, glycosyl transferases (GTs) showed the main difference (Figure 8B).

## Discussion

This study revealed that the bacterial OTU richness of the three bamboo species in autumn was significantly higher than that in spring. The Phyllosphere of *F. ferax* had a greater diversity of bacterial OTUs than that of *A. spanostachya* and *Y. lineolate*. The dominant phyllosphere bacteria of the three bamboo species are Proteobacteria, Acidobacteria, Bacteroides, and Actinomycetes. In a warm and humid climate, the diversity and richness of phyllosphere bacteria in the three bamboo species in spring were significantly higher than that in autumn. The overall importance of seasonality to the structure and composition of the phyllosphere microbial community has been confirmed by many studies (Thompson et al., 1993; Copeland et al., 2015). Proteobacteria, Acidobacteria, Bacteroides, and Actinomycetes are often detected in a variety of forests, indicating that these organisms have a wide ecological range and an ability to adapt to many environments (Laforest et al., 2016; Feng et al., 2019). In this study, the relative abundance of the Proteobacteria in the three bamboo species was all above 60%, and the differences between the bamboo species were not significant, indicating that Proteobacteria played a dominant role in the phyllosphere microbial community. Its variation in turn may impact the health of the staple food bamboo foraged by giant panda around Xiaoxiangling mountains.

The bacterial diversity and abundance of all three bamboo species in autumn were significantly higher than that in spring, which was similar to the result of Zheng (2011), who found that the number of phyllosphere microbial communities of *Pinus tabulaeformis* varied significantly between different seasons, with the largest diversity and abundance in autumn, followed by summer and the least in spring. Higher temperatures and humidity in summer and autumn resulted in higher species diversity and richness than in spring, while higher elevations and longer periods of low temperatures in winter could result in lower species diversity and richness than in spring (Laforest et al., 2016).

The Mantel test found that elevation, distance from water, tree diameter at breast height, mean height of bamboo, mean base diameter of bamboo, tree height, shrub coverage, and the number of shrubs all significantly affected the phyllosphere microbial community (Supplementary Table 3). Jackson et al. (2010) found that the changes in the phyllosphere bacterial community in resurgent ferns were related to rainfall and humidity. Similarly, Laforest et al. (2016) found that the host species, habitat, and climate (average summer temperature and precipitation) drove the phyllosphere bacterial community structure in temperate trees. In this study, elevation had the strongest relationship with phyllosphere microbial community. Elevation was significantly and positively correlated to many bacteria phyla abundance, such as Proteobacteria (Figure 6). However, with an increase in elevation, both the Shannon and Sobs indices declined (Supplementary Figure 6). Higher elevation generally resulted in lower temperature, which can limit the fluidity of microbial cell membranes and proteins, which is not conducive to microbial reproduction and growth (Zhang et al., 2014).

Changes in the phyllosphere microbial community could impact the degradation and absorption of plant nutrients and the metabolism of enzymes (Fazal et al., 2021). In this study, we used PICRUSt to predict the function of phyllosphere bacteria of three bamboo species foraged by giant pandas. Our results have shown that the relative abundance of gene function in the membrane transport secretion system, signal transduction, and oxidative phosphorylation metabolism in the third level of the KEGG pathway for *F. ferax* was lower than that of the other two bamboo species (Figure 7). This implied that some phyllosphere bacteria flora in *F. ferax* at lower altitude need less metabolic activity to maintain the physiological activities relative to that in high-altitude *A. spanostachya* and *Y. lineolate* phyllosphere. It is worth noting that the relative abundances of gene functions about transporters and ABC transporters in the bamboo phyllosphere bacterial community were the most expressed pathways in membrane transport. Hamana et al. (2012) revealed that ABC transporters can protect animals from the barrier of toxic substances. In addition, genes related to replication and repair may help reduce damage to biomolecules and may help bamboo adapt to high-altitude environments.

Different microbial compositions, diversity, and community structure imply different functional characteristics. Further metagenomic analysis uncovered the significant differences in biological functions (KEGG and Carbohydrate-Active enzymes functions) of *A. spanostachya* phyllosphere bacteria between spring and autumn (Figure 8). In spring, despite the pathways of human diseases (infectious disease: bacterial and viral, cancer: overview and specific types, cardiovascular disease, and drug resistance: antineoplastic) being more abundant, the raised biological functions including the immune system and biosynthesis of other secondary metabolites in autumn may neutralize these negative effects. Abundant xenobiotics biodegradation and metabolism function be conducive to *A. spanostachya* phyllosphere health. More abundant nucleotide metabolism, glycan biosynthesis and metabolism, and cellular community indicated stronger bacterial cell viability and cell proliferation in autumn. The LEfse results of the CAZyme support an important role for the metabolism of carbohydrates in bacterial survival in the plant phyllosphere (Ryffel et al., 2015; Müller et al., 2016). GHs families can break down complex carbohydrates (Lee et al., 2013) and played a crucial role in the processing of various exogenous and endogenous glycoconjugate in human gut microbiota (Pellock et al., 2018). Our results father indicated a diversification in carbohydrate metabolism pathways existing in *A. spanostachya* phyllosphere bacterial microorganisms in spring, which may be due to harsh environment, such as low temperatures in lengthy winter (Laforest et al., 2016).

Food microbes can affect the gut microbes of animals (Kohl and Dearing, 2014; Kohl et al., 2017). Jin et al. (2020) found significant associations of certain bacteria and fungi between bamboo and the gut of giant panda. The diversity of bamboo bacteria was also positively correlated with that of gut bacteria in giant panda. Giant pandas prefer to consume bamboo that grows naturally at high altitudes, probably because the total number of endophytic bacteria in high altitudes tends to be lower (Helander et al., 2013). There is a lot of work needed to fully understand the relationship between the food microbes and gut microbes of pandas. To explore the relevance of these genes and their functions in the environmental adaptability of giant pandas and bamboo, further research is needed to determine whether there are specific enzymes coded in phyllosphere microorganisms related to digestion in giant pandas’ gut.

## Conclusion

In this study, high-throughput sequencing was used to explore phyllosphere bacterial communities in three bamboo species (*A. spanostachya*, *Y. lineolate*, and *F. ferax*) foraged by giant panda in different seasons (spring vs. autumn), in Liziping National Nature Reserve (Liziping NR), China. Our findings suggested that the diversity of *F. ferax* phyllosphere bacterial species was greater than that of the other two bamboo species in both seasons, indicating that low altitude may be a promoter of bamboo phyllosphere microbial richness and diversity, of course, strong human interference at low altitudes was also a factor. Besides, phyllosphere bacterial diversity was also significantly higher in autumn than in spring, implying that season-related changes in environmental factors (e.g., temperature and moisture) may influence bacterial communities. In autumn, there was a lower abundance and diversity of phyllosphere bacteria at higher altitudes, and giant pandas chose more high-altitude bamboo species like *A. spanostachya* as their staple food. In spring, a lower abundance and diversity of bamboo phyllosphere bacteria was found, and giant pandas chose some low-altitude bamboo species like *Y. lineolate* as their staple food. Long-term low temperatures may shape more varied and complex KEGG and Carbohydrate-Active enzymes functions in spring. Interestingly, the feeding of giant pandas on bamboo species was consistent with low bacterial concentrations in bamboo leaves. Was this related to the giant panda gut microbes’ adaptability? This needs rigorous controlled experimental approaches to investigate. All these findings may provide a reference for the restoration and management of giant panda bamboo dietary and food resources in this area, especially for those small isolated populations of giant pandas in Xiaoxiangling mountains.

## Data Availability Statement

The datasets presented in this study can be found in online repositories. The names of the repository/repositories and accession number(s) can be found below: https://www.ncbi.nlm.nih.gov/, PRJNA718425.

## Author Contributions

MH, ZZ, and JL conceived and designed the study. JL, WL, JX, YY, JW, LK, and YL collected data and samples in the field. MH, JL, JX, YY, and JW processed samples in the lab. MH, JL, and YY analyzed the data. MH, ZZ, JL, and LW wrote the manuscript. All authors read and approved the final manuscript.

## Conflict of Interest

The authors declare that the research was conducted in the absence of any commercial or financial relationships that could be construed as a potential conflict of interest.

## Publisher’s Note

All claims expressed in this article are solely those of the authors and do not necessarily represent those of their affiliated organizations, or those of the publisher, the editors and the reviewers. Any product that may be evaluated in this article, or claim that may be made by its manufacturer, is not guaranteed or endorsed by the publisher.

## Footnotes

1
https://github.com/OpenGene/fastp

2
http://bio-bwa.sourceforge.net

3
https://github.com/voutcn/megahit

4
http://www.ncbi.nlm.nih.gov/Taxonomy/taxonomyhome.html/index.cgi?chapter=tgencodes#SG1

5
http://www.bioinformatics.org/cd-hit/

6
https://github.com/bbuchfink/diamond

7
http://www.genome.jp/kegg/

8
http://www.cazy.org/
