# Supplementary material for: Environmental Factors Influencing Phyllosphere Bacterial Communities in Giant Pandas’ Staple Food Bamboos
Source: Front Microbiol. 2021 Nov 3;12:748141. doi: 10.3389/fmicb.2021.748141 (PMC8595598; doi:10.3389/fmicb.2021.748141)
Supplement: Supplementary file 1 [file Data_Sheet_1.zip › Supplementary Figures.docx]

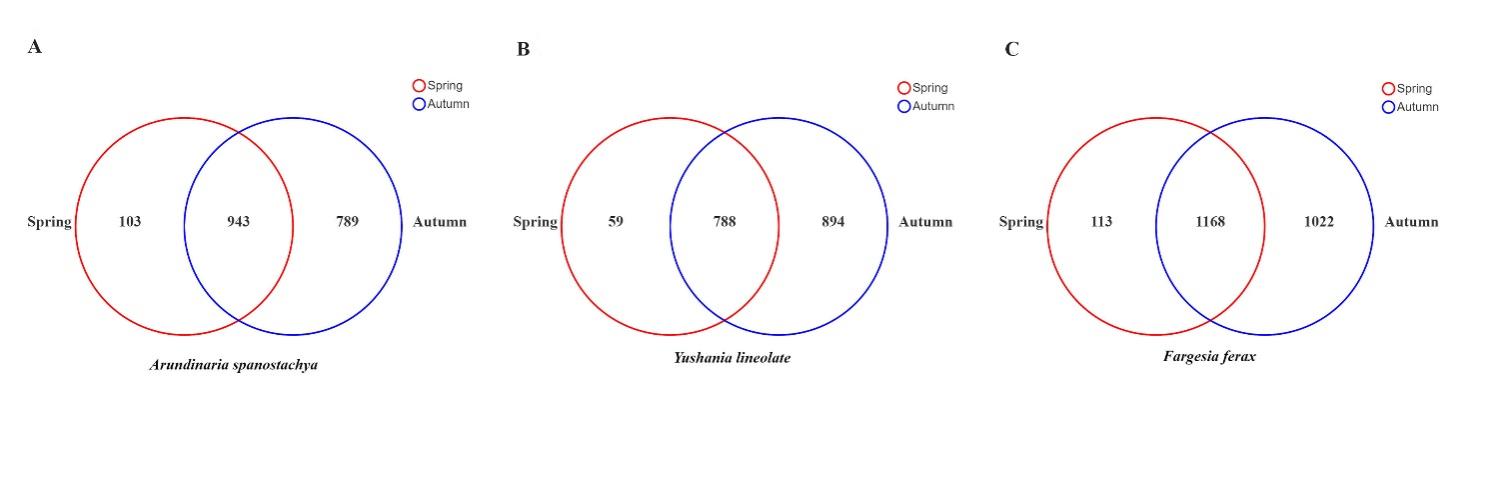


**Supplementary Figure 1.** Venn Diagram showing overlap/non-overlap of phyllosphere bacteria OTUs of *A. spanostachya* (AS) (**A**), *Y. lineolate* (YL) (**B**) and *F. ferax* (FF) (**C**) between spring and autumn.


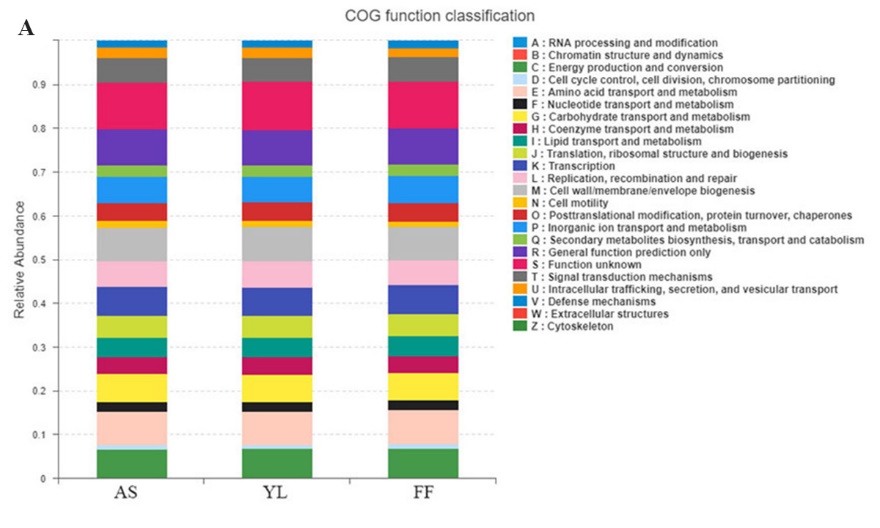

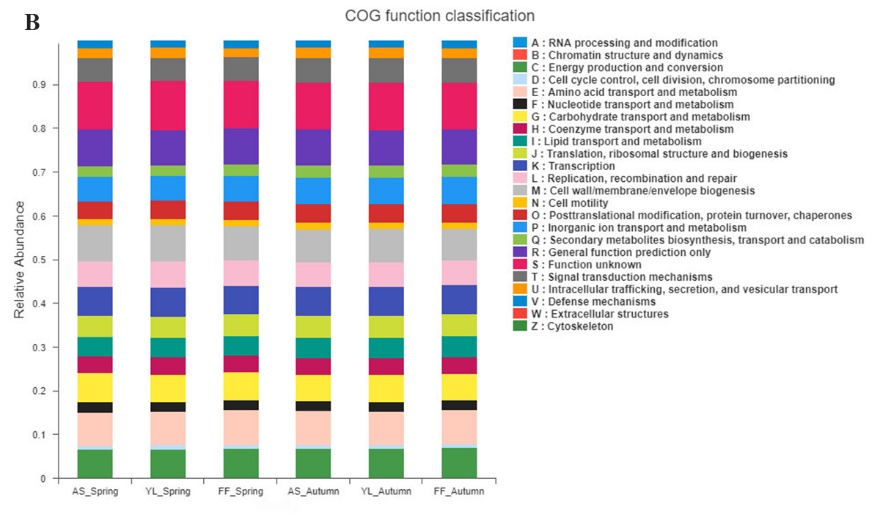


**Supplementary Figure 2.** Histogram of COG functional classification of phyllosphere bacteria among *A. spanostachya* (AS), *Y. lineolate* (YL) and *F. ferax* (FF) (**A**) in spring and autumn (**B**).
