# Supplementary material for: Environmental Factors Influencing Phyllosphere Bacterial Communities in Giant Pandas’ Staple Food Bamboos
Source: Front Microbiol. 2021 Nov 3;12:748141. doi: 10.3389/fmicb.2021.748141 (PMC8595598; doi:10.3389/fmicb.2021.748141)
Supplement: Supplementary file 1 [file Data_Sheet_1.zip › Supplementary Tables/Table S1, Table S2, Table S3, Table S4, Table S5, Table S7.docx]

**Table S1.** Definition and measurement of each variable.

| Variables(Units) | Definition and Measurement |
| --- | --- |
| Elevation, E (m) | Elevation of central point of habitat quadrat (20×20m^2^) |
| Slope, S (°) | Slope of central point of habitat quadrat (20×20m^2^) |
| Water source distance, DW (m) | Distance from center point of habitat quadrat (20×20m^2^) to visible or audible water flow |
| Tree height, TH (m) | Average height of trees over 5m in a habitat quadrat (20×20m^2^) |
| Tree DBH, TDBH (cm) | Mean DBH of trees over 5m in habitat quadrat (20×20m^2^) |
| Number of trees, NT | Number of trees over 5m in habitat quadrat (20×20m^2^) |
| Canopy density, CD (%) | Canopy density of trees over 5m in habitat quadrat (20×20m^2^) (%) |
| Shrub height, SH (m) | Average height of shrubs no more than 5m high in habitat square (20×20m^2^) |
| Shrub coverage, SC (%) | Mean DBH of shrubs no more than 5m high in habitat square (20×20m^2^) |
| Shrubs number, SN | Number of shrubs no more than 5m high in habitat square (20×20m^2^) |
| Bamboo coverage, BC (%) | Average bamboo coverage (%) of 3 bamboo quadrats (1×1m^2^) |
| Total number of live bamboo, TNLB | Average number of live bamboo in 3 bamboo quadrats (1×1m^2^) |
| Bamboo deaths, BD | The average number of dead bamboo in 3 bamboo quadrats (1×1m^2^) |
| Annual number of bamboo | Average annual bamboo number of 3 bamboo quadrats (1×1m^2^) |
| Annual bamboo height (cm) | The 3 bamboo quadrats (1×1m^2^) averaged annual bamboo height |
| Annual bamboo base Diameter (mm) | Three bamboo quadrats (1×1m^2^) averaged annual bamboo base |
| Number of biennial bamboo | 3 bamboo quadrats (1×1m^2^) mean biennial bamboo quantity |
| Biennial bamboo height (cm) | The average height of the 3 bamboo quadrats (1×1m^2^) was 2 years |
| Biennial bamboo base diameter (mm) | Three bamboo quadrats (1×1m^2^) averaged biennial growth of bamboo base meristem |
| Number of perennial bamboo | Average number of perennials of 3 bamboo quadrats (1×1m^2^) |
| Perennial bamboo height (cm) | Three bamboo quadrats (1×1m^2^) had average perennial bamboo height |
| Perennial bamboo base diameter (mm) | Three bamboo quadrats (1×1m^2^) averaged perennial bamboo base meristem |

**Table S2.** The observed species index (Sobs index) and the Shannon index of phyllosphere bacterial community among different seasons and bamboo species. (The data in the table is the mean±standard deviation).

| Diversity index | Autumn | | | Spring | | |
| --- | --- | --- | --- | --- | --- | --- |
|  | *Fargesia*  *ferax* | *Yushania lineolate* | *Arundinaria spanostachya* | *Fargesia ferax* | *Yushania lineolate* | *Arundinaria spanostachya* |
| Shannon index | 5.09±0.43 | 4.92±0.26 | 4.73±0.27 | 4.26±0.33 | 3.82±0.31 | 4.16±0.28 |
| Sobs index | 798.62±174.12 | 651.44±91.88 | 596.05±103.07 | 444.25±93.82 | 308.12±54.83 | 387.35±66.79 |

**Table S3.** Mantel tests exploring the association between each environmental factor distance matrix and phyllosphere bacterial UniFrac distance matrix based on OUT level.

| Environmental factors | R | p |
| --- | --- | --- |
| Elevation, E | 0.315 | **0.001** |
| Slope, S | 0.007 | 0.838 |
| Water source distance, DW | 0.162 | **0.001** |
| Tree height, TH | 0.083 | **0.032** |
| Tree DBH, TDBH | 0.134 | **0.002** |
| Number of trees, NT | 0.052 | 0.121 |
| Canopy density, CD | -0.033 | 0.395 |
| Shrub height, SH | 0.031 | 0.512 |
| Shrub coverage, SC (%) | 0.093 | **0.003** |
| Shrubs number, SN | 0.07 | **0.005** |
| Bamboo coverage, BC | 0.08 | 0.054 |
| Total number of live bamboo, TNLB | 0.008 | 0.843 |
| Bamboo deaths, BD | 0.053 | 0.210 |
| Mean height of bamboo, MHB | 0.122 | **0.001** |
| Mean base diameter of bamboo, MBDB | 0.175 | **0.001** |

**Table S4.** RDA analysis detecting the relationship between each environmental factor and phyllosphere bacterial community in each sample based on OUT level.

| Environmental factors | RDA1 | RDA2 | R^2^ | p |
| --- | --- | --- | --- | --- |
| Elevation, E | -0.3395 | -0.9406 | 0.2561 | **0.001** |
| Slope, S | -0.9938 | -0.1109 | 0.0019 | 0.915 |
| Water source distance, DW | -0.2053 | 0.9787 | 0.0033 | 0.856 |
| Tree height, TH | 0.83 | 0.5578 | 0.08 | **0.015** |
| Tree DBH, TDBH | 0.6794 | -0.7337 | 0.1168 | **0.004** |
| Number of trees, NT | -0.0941 | 0.9956 | 0.0854 | **0.012** |
| Canopy density, CD | -0.116 | 0.9932 | 0.1499 | **0.001** |
| Shrub height, SH | 0.7373 | -0.6756 | 0.0089 | 0.611 |
| Shrub coverage, SC | 0.945 | -0.3272 | 0.0887 | 0.013 |
| Shrubs number, SN | 0.9273 | 0.3744 | 0.0693 | **0.040** |
| Bamboo coverage, BC | -0.9987 | 0.0508 | 0.057 | **0.046** |
| Total number of live bamboo, TNLB | 0.9851 | 0.1717 | 0.0459 | 0.099 |
| Bamboo deaths, BD | 0.9331 | 0.3597 | 0.1947 | **0.001** |
| Mean height of bamboo, MHB | -0.1675 | 0.9859 | 0.2878 | **0.001** |
| Mean base diameter of bamboo, MBDB | 0.3195 | 0.9476 | 0.4121 | **0.001** |

**Table S5.** Linear Regression between environmental factors and Sobs index and Shannon index, and Bray-Curtis distance matrixes. Factors associated with P values lower than 0.05 are in bold font.

| Ecology factors | Sobs | | | Shannon | | | Bray-Curtis distance | | |
| --- | --- | --- | --- | --- | --- | --- | --- | --- | --- |
|  | F | R² | p | F | R² | p | F | R² | p |
| Elevation, E | **17.299** | **0.149** | **0.000** | **7.648** | **0.072** | **0.007** | **10.214** | **0.094** | **0.002** |
| Bamboo deaths, BD | **16.940** | **0.146** | **0.000** | **20.995** | **0.175** | **0.000** | **20.394** | **0.171** | **0.000** |
| Mean base diameter of bamboo, MBDB | **15.775** | **0.137** | **0.000** | **8.094** | **0.076** | **0.005** | **10.148** | **0.093** | **0.002** |
| Bamboo coverage, BC | **8.590** | **0.080** | **0.004** | **4.456** | **0.043** | **0.037** | **7.975** | **0.075** | **0.006** |
| Tree height, TH | **6.567** | **0.062** | **0.012** | **5.272** | **0.051** | **0.024** | **7.295** | **0.069** | **0.008** |
| Shrub coverage, SC | 3.670 | 0.036 | 0.058 | 3.049 | 0.030 | 0.084 | **6.594** | **0.062** | **0.012** |
| Shrubs number, SN | 2.706 | 0.027 | 0.103 | 2.779 | 0.027 | 0.099 | **6.080** | **0.058** | **0.015** |
| Water source distance, DW | 1.544 | 0.015 | 0.217 | 0.648 | 0.007 | 0.423 | 0.356 | 0.004 | 0.552 |
| Tree DBH, TDBH | 1.494 | 0.015 | 0.225 | 3.602 | 0.035 | 0.061 | 1.735 | 0.017 | 0.191 |
| Number of trees, NT | 0.974 | 0.010 | 0.326 | 0.073 | 0.001 | 0.787 | 0.344 | 0.004 | 0.559 |
| Canopy density, CD | 0.777 | 0.008 | 0.380 | 0.001 | 0.000 | 0.971 | 0.002 | 0.000 | 0.967 |
| Total number of live bamboo, TNLB | 0.668 | 0.007 | 0.416 | 3.653 | 0.036 | 0.059 | 2.465 | 0.024 | 0.120 |
| Shrub height, SH | 0.445 | 0.005 | 0.506 | 0.127 | 0.001 | 0.722 | 0.228 | 0.002 | 0.634 |
| Mean height of bamboo, MHB | 0.442 | 0.004 | 0.508 | 0.013 | 0.000 | 0.911 | 0.015 | 0.000 | 0.904 |
| Slope, S | 0.279 | 0.003 | 0.599 | 0.130 | 0.001 | 0.719 | 0.008 | 0.000 | 0.928 |

**Table S7.** Metagenomic sequences of *A. spanostachya* samples.

| Seasons | Sample ID | Raw reads | Clean reads | Q30 (%) | Optimized reads | Contigs | Assembly Length(bp) | N50(bp) |
| --- | --- | --- | --- | --- | --- | --- | --- | --- |
| Spring | AS_2 | 47323768 | 46155256 | 93.22 | 19017874 | 240139 | 139860895 | 594 |
|  | AS_5 | 52429806 | 51370938 | 94.11 | 33380788 | 422705 | 290630012 | 706 |
|  | AS_6 | 56462214 | 55290156 | 94.19 | 42023322 | 548051 | 396099576 | 785 |
|  | AS_11 | 51167404 | 50228146 | 94.47 | 37621446 | 454143 | 331030840 | 799 |
| Autumn | AS_23 | 58458226 | 57332592 | 93.79 | 47354156 | 399195 | 213032834 | 536 |
|  | AS_32 | 52615294 | 50213930 | 94.15 | 42259996 | 538727 | 303451597 | 566 |
|  | AS_35 | 57218662 | 55556410 | 93.83 | 49497140 | 678879 | 415232248 | 624 |
|  | AS_36 | 55994632 | 54837284 | 93.94 | 49475978 | 631829 | 384565815 | 628 |
